# Pathogenicity and Transmissibility of North American H7 Low Pathogenic Avian Influenza Viruses in Chickens and Turkeys

**DOI:** 10.3390/v11020163

**Published:** 2019-02-16

**Authors:** Ishita Roy Chowdhury, Sai Goutham Reddy Yeddula, Shin-Hee Kim

**Affiliations:** VA-MD Regional College of Veterinary Medicine, University of Maryland, College Park, MD 20742, USA; iroychow@umd.edu (I.R.C.); yeddulas@umd.edu (S.G.R.Y.)

**Keywords:** low pathogenic avian influenza virus, H7, replication, transmissibility, chickens

## Abstract

Low pathogenic avian influenza (LPAI) viruses can silently circulate in poultry and wild aquatic birds and potentially mutate into highly pathogenic avian influenza (HPAI) viruses. In the U.S., recent emergence and spread of H7N8 and H7N9 HPAI viruses not only caused devastating losses to domestic poultry but also underscored the capability of LPAI viruses to mutate into HPAI viruses. Therefore, in this study, we evaluated pathogenicity and transmissibility of H7N8 and H7N9 LPAI viruses (the progenitors of HPAI viruses) in chickens and turkeys. We also included H7N2 isolated from an outbreak of LPAI in commercial chickens. H7 viruses replicated more efficiently in the respiratory tract than in the gastrointestinal tract, suggesting that their replication is restricted to the upper respiratory tract. Specifically, H7N2 replicated most efficiently in two-week-old chickens and turkeys. In contrast, H7N8 replicated least efficiently in those birds. Further, replication of H7N2 and H7N9 was restricted in the upper respiratory tract of four-week-old specific-pathogen-free (SPF) and broiler chickens. Despite their restricted replication, the two viruses efficiently transmitted from infected to naïve birds by direct contact, leading to seroconversion of contacted chickens. Our findings suggest the importance of continuous monitoring and surveillance of LPAI viruses in the fields.

## 1. Introduction

Avian influenza virus (AIV) belongs to the family *Orthomyxoviridae* and the genus *Influenzavirus* A. The virus has a negative-sense, single-stranded and segmented RNA genome and contains eight gene segments encoding at least 10 proteins: Polymerase basic 1 (PB1), PB2, polymerase acid (PA), hemagglutinin (HA), nucleoprotein (NP), neuraminidase (NA), matrix 1 (M1), M2, nonstructural 1 (NS1) and 2 (NS2) [1]. The natural reservoirs of the virus are wild aquatic birds with ducks, gulls and shorebirds being the primary hosts, which can result in the wide geographic spread and distribution of circulating viruses [2]. On the basis of antigenic specificity, 16 HA types and 9 NA types have been detected in viruses isolated from wild waterfowl. 

Most AIVs cause little to no disease in birds. These low pathogenic avian influenza (LPAI) viruses contain an HA cleavage site which can only be cleaved by proteases available in the intestinal and respiratory tracts [3]. In contrast, highly pathogenic avian influenza (HPAI) viruses contain multiple basic amino acids at the HA0 cleavage site, resulting in cleavability of HA by ubiquitous intracellular proteases. Therefore, HPAI viruses cause systemic infection and high mortality in chickens and other terrestrial poultry. Two subtypes (H5 and H7) of LPAIVs can naturally switch to a highly pathogenic phenotype through mechanisms, such as acquisition of basic amino acids in the cleavage region of the HA protein by insertion or substitution and recombination with another gene segment(s) or host genome [4,5]. 

H7 subtype viruses are widely distributed and cause high mortality in gallinaceous poultry with substantial economic losses for the poultry industry and sporadic human infections [6,7]. LPAI viruses are the precursors of numerous outbreaks of HPAI viruses in commercial poultry farms. Specifically, H7N1 HPAI was evolved from an LPAI precursor and caused the death of over 16 million poultry and substantial economic losses to industry in Northern Italy (1999–2001) [8]. In 2008, mutation of H7N7 LPAIV to HPAIV occurred in free range laying hens during an outbreak in Oxfordshire, U.K. [9]. Phylogenetic analysis indicated incursion of a wild bird origin LPAIV precursor to the H7N7 HPAIV outbreak during 2006–2008.

H7 HPAI viruses have emerged multiple times in commercial poultry in the U.S. [10]. In 2016, H7N8 HPAIV caused an outbreak in turkeys in Indiana [11]. Subsequently, a progenitor LPAI virus was detected in turkey flocks during control zone surveillance. Sequence analysis indicated that the HPAI was evolved from the LPAI circulated among diving ducks in the Mississippi flyway [12]. The H7N8 virus was eradicated from Indiana after quarantine and depopulation of 414,223 commercial birds, including 258,045 turkeys and 156,178 chickens, on 10 commercial turkey farms. In 2017, concurrent outbreaks of H7N9 HPAI and LPAI were occurring at poultry farms in Tennessee. Subsequently, H7N9 LPAI were detected in additional commercial and backyard flocks in Alabama, Kentucky, and Georgia. More than 270,000 birds died or were culled. Genetic analyses have identified the virus in the Wyoming blue-winged teal as a precursor to the poultry isolates. The virus had been silently circulated in the region, indicating a spillover of circulating LPAI into commercial poultry. This LPAI virus mutated to HPAI virus through acquisition of basic amino acids at the cleavage site of the HA protein by recombination with chicken rRNA gene during virus replication in chickens [13]. 

The detection and spread of H5 and H7 LPAI viruses in gallinaceous poultry are considered as an indicative emergence of HPAI viruses. This highlights the importance of controlling LPAI viruses in the field and need for routine and frequent testing of poultry for AIV. For a better control of LPAI viruses, we evaluated replication and bird-to-bird transmissibility of North American H7N8 and H7N9 LPAI viruses that were isolated from commercial poultry farms during AI outbreaks in 2016 and 2017, respectively. In this study, we included one of the H7N2 isolates from commercial chicken farms in 2004.

## 2. Materials and Methods 

### 2.1. Viruses 

LPAI viruses used in this study were A/Chicken/Delaware/VIVA/04 (H7N2), A/TY/IN/1573-2/2016 (H7N8), and A/chicken/Tennessee/17-007431-3/2017 (H7N9). H7N2 was isolated from a commercial broiler chicken in Delaware during outbreaks of AIV in 2004 [14]. H7N8 and H7N9 were obtained from the National Veterinary Services Laboratory (Ames, IA, USA). The viruses were grown in 9-day-old specific pathogen free (SPF) embryonated chicken eggs. Since the hemagglutination assay (HA) titers of H7N8 and H7N9 were low (<2^6^), the viruses were additionally passaged in the eggs. The HA titers of the two viruses were increased to 2^8^ after the third passage in eggs. After each passage of the viruses, we conducted nucleotide sequence analyses of the HA and NA genes by RT-PCR and did not detect occurrence of any mutations during viral passages. 

### 2.2. Cytopathic Effect of H7 Viruses and the HA Cleavability in MDCK Cells

To compare their cytopathic effect (CPE), Madin–Darby canine kidney (MDCK) cells in a 6 well plate were individually infected with H7N2, H7N8, and H7N9 at a multiplicity of infection (MOI) of 0.1 PFU/cell. After 1 h of adsorption, the culture medium was changed with Dulbecco’s modified Eagle’s medium (DMEM) with or without the addition of N-tosyl-l-phenylalanine chloromethyl ketone (TPCK)-treated trypsin (1 μg/mL). After 12 h post-infection (hpi), the infected cells were visualized directly by photomicroscopy. Further, efficient cleavage of HA protein in virus-infected MDCK cells was evaluated by Western blot analysis. Cell lysates were separated by SDS-PAGE, and transferred protein into a nitrocellulose membrane was detected by using anti-H7 HA, A/Netherlands/219/2003 (H7N7) (BEI resources). Plaque formation of H7 viruses in MDCK cells was conducted by plaque assay [15]. 

### 2.3. Replication of H7 Viruses in 2-Week-Old Chickens and Turkeys

Replication of H7N2, H7N8, and H7N9 viruses was determined in 2-week-old chickens (SPF) and turkeys. One-day-old turkeys (White Holland) were obtained from Murray McMurray farm (Webster City, IA) and housed in our enhanced BSL-2 (ABSL-2) facility. All turkeys were confirmed to be seronegative to H7 viruses. Birds (*n* = 6 for each virus) were intranasally inoculated with 200 µL of each virus (10^7^ pfu/mL). Tissue samples (lung, trachea, spleen, and brain) were collected from three birds for virus titration in MDCK cells on days 3 post-infection (pi). Collected samples were homogenized, diluted, and inoculated into MDCK cells. Virus titers were expressed as 50% tissue culture infectious dose (TCID_50_/g) by the end-point method of Reed and Muench [16]. In addition, swab (oral and cloacal) samples were collected for virus shedding at 3 days post-infection (dpi). Individual swab samples were placed in a tube containing 1 mL of DMEM culture medium with antibiotics. Viral shedding was confirmed by replication of the virus in MDCK cells. Supernatants (500 µL each) of oral and cloacal samples were inoculated into MDCK cells in a 12-well plate. At 72 h post-infection (hpi), viral replication was determined by HA assay. The remaining birds were observed daily up to 14 dpi for any clinical signs. Seroconversion was determined by HI assay. All of the animal experiments were conducted in ABSL-2 facility following the guidelines and approval of the Animal Care and Use Committee (IACUC) (R-JUN-17-28, 20/06/2017) and Institutional Biosecurity Committee (IBC), University of Maryland.

### 2.4. Replication and Transmission of H7N2 and H7N9 Viruses in 4-Week-Old SPF and Broiler Chickens 

Replication and transmissibility of H7N2 and H7N9 viruses were determined in four-week-old SPF (specific-pathogen-free) and broiler chickens. One-day-old broiler chickens were obtained from a commercial hatchery (Amick Farm Hurlock Hatchery, Hurlock, MD, USA) and housed in ABSL-2 facility. All chickens were seronegative to H7 viruses. Chickens (*n* = 6 for each virus) were intranasally inoculated with 200 µL of each virus (10^7^ pfu/mL). On days 3 pi, tissue samples (lung and trachea) were collected from three chickens for virus titration in MDCK cells. In addition, trachea samples were fixed in phosphate-buffered formalin (10%), processed for section and stained with hematoxylin and eosin. Sections from mock-infected birds were used as controls.

In parallel with viral replication, transmissibility of H7 viruses was determined in SPF and broiler chickens. Each experiment, virus-infected birds (*n* = 3) were placed in an isolator. At 24 dpi, three contact exposure birds (naïve birds) of the same species were placed into the same isolator. After the first day of exposure, swab (oral and cloacal) samples were collected daily up to 5 days, and virus shedding was determined as described above. Birds were monitored daily for clinical signs up to 14 days post-exposure (dpe). Seroconversion was determined by HI assay.

### 2.5. Statistical Analysis

Statistically significant differences in replication of H7 viruses in vivo were evaluated by one-way analysis of variance (ANOVA) using the Turkey’s multiple comparison test. All of the results were analyzed using Prism 5.0 (GraphPad Software Inc., San Diego, CA, USA) with a significance level of *p* < 0.05.

## 3. Results

### 3.1. In Vitro Characterization of H7 Viruses 

**Figure 1:**
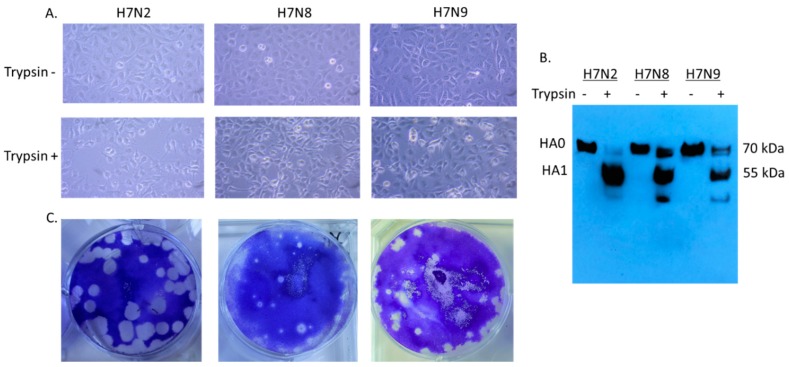
Infectivity of H7N2, H7N8, and H7N9 in MDCK (Madin–Darby canine kidney) cells. MDCK cells were individually infected with H7N2, H7N8, and H7N9 (MOI of 0.1 PFU/cell) in the presence or absence of trypsin. After 12 hpi, the infected cells were visualized directly by photomicroscopy, (20X) (**A**). Collected cell lysates were subjected to Western blot analysis (**B**). Plaque formation of H7 viruses in MDCK cells was conducted by plaque assay (**C**).

Since H7N8 and H7N9 strains are recently emerged viruses, little information is available for viral infectivity. We first evaluated cytopathic effect (CPE) in virus-infected cells (Figure 1A). In 12 hpi, syncytium formation was detected in virus-infected cells in the presence of trypsin. H7N2 produced more extensive syncytia than H7N8 and H7N9. Proteolytic cleavage of the HA protein in mature virus particles is critical for virus infectivity and spread of the virus through cell-cell fusion (1). Therefore, the cell lysates were collected to evaluate infectivity of H7 viruses. We included cell lysates without addition of trypsin as a control. Cleavage of HA protein was detected in MDCK cells in the presence of trypsin (Figure 1B). In H7N2 infected cells, cleaved HA1 protein (55 kDa) was dominant compared to uncleaved HA0 (70 kDa), indicating efficient cleavage of HA protein. As expected, only uncleaved HA protein was detected in the infected cells without addition of trypsin. All three viruses produced plaques in the presence of trypsin (Figure 1C). H7N2 produced the most distinguishable size of plaque in MDCK cells followed by H7N9. This in vitro characterization suggested that H7N2 can be more infectious than H7N8 and H7N9.

### 3.2. Replication of H7 Viruses Was Restricted to Upper Respiratory Tract in 2-Week-Old Chickens and Turkeys

**Figure 2:**
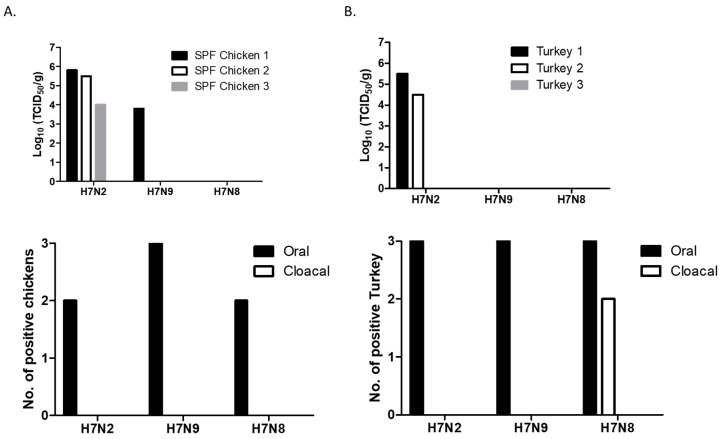
Replication and shedding of H7 LPAI viruses in the trachea of 2-week-old SPF (specific-pathogen-free) chickens (**A**) and turkeys (**B**). Birds were intranasally inoculated with 200 µL of each virus (10^7^ pfu/mL). Trachea samples were collected for virus titration in MDCK cells on days 3 pi. Virus titers were expressed as 50% tissue culture infectious dose (TCID_50_/g). Swab (oral and cloacal) samples were collected for virus shedding. Viral replication was confirmed by amplifying the virus in MDCK cells. At 72 h post-infection, viral replication was determined by HA assay.

Virus replication was determined in chickens and turkeys, two major poultry associated with the outbreaks of AIV. In SPF chickens, replication of H7 viruses was mostly restricted to the trachea. Virus replication was not detected in lungs (except one chicken), spleens, and brain of infected chickens. H7N2 replicated well in the trachea of all three chickens (Figure 2A). One chicken also replicated in the lung (2 × 10^4^ TCID_50_/g). H7N9 replicated in the trachea of one chicken. The titer of H7N9 was lower than those of H7N2 infected chickens. In contrast, replication of H7N8 was not detected in the collected tissue samples. In general, shedding of H7 viruses was only detected in oral swab samples at 3 dpi, indicating that viral replication was limited to the upper respiratory tract.

In turkeys, H7N2 replicated in the trachea of two birds (Figure 2B). The viral titers of the trachea between chickens and turkeys did not show significant difference. We did not detect replication of H7N8 and H7N9 in the trachea of turkeys. Furthermore, H7 viruses did not replicate in the lungs, spleens, and brains. Their shedding was detected in oral swabs collected from all the infected turkeys, indicating that the viruses replicated mostly in the site of inoculation. Interestingly, shedding of H7N8 (a turkey isolate) was detected in cloacal swabs collected from two turkeys.

All the remaining chickens and turkeys were healthy without exhibiting any clinical signs up to 14 dpi. Despite their restricted replication, we found seroconversion of all the chickens and turkeys infected with H7N2 or H7N9 (HI titer >2^5^). In H7N8 infected groups, seroconversion was found in turkeys (HI titer 2^6^). In contrast, significantly low HI titers was detected in the chickens infected with H7N8, suggesting that H7N8 strain may not be adapted for efficient replication in chickens. This in vivo replication result suggests inefficient replication of H7N8 in poultry. Therefore, we further compared replication and transmissibility of only two H7 viruses (H7N2 and H7N9) in 4-week-old chickens. 

### 3.3. Attenuation of H7N9 in 4-Week-Old SPF and Broiler Chickens 

**Figure 3:**
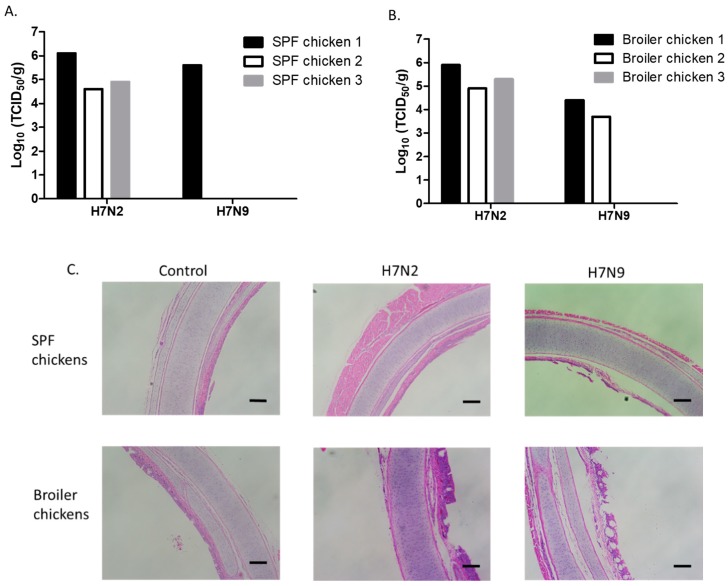
Replication of H7N2 and H7N9 in the trachea of 4-week-old SPF and broiler chickens. Birds were intranasally inoculated with 200 µL of each virus (10^7^ pfu/mL). Trachea samples were collected from SPF (**A**) and broiler (**B**) chickens for virus titration in MDCK cells on days 3 pi. Virus titers were expressed as 50% tissue culture infectious dose (TCID_50_/g). For histopathology, trachea samples were collected from uninfected or infected 4-week-old SPF and broiler chickens (**C**). The samples were fixed in phosphate-buffered formalin, were processed for section, and stained with hematoxylin and eosin (scale bar = 2 mm).

Replication of H7N2 and H7N9 was evaluated by infecting 4-week-old SPF (Figure 3A) and broiler chickens (Figure 3B). We found a similar pattern in the replication of H7N2 and H7N9 viruses between 2-week- and 4-week-old SPF chickens. Further, the titers of H7N2 in the trachea of 4-week-old SPF and broiler chickens were not significantly different (Figure 3A,B). H7N9 replicated in the trachea of one SPF and two broiler chickens. We further determined the pathology of the trachea samples (Figure 3C). Cilia loss and heterophilic inflammation were mostly detected in the trachea of infected chickens. In general, thickening of the mucosa was mostly detected in the trachea of broiler chickens. Distorted mucous glands were also found in the trachea infected with H7N2. These results suggest that H7N2 not only replicated better than H7N9 but also induced distinguishable histological lesions in the trachea. H7N2 and H7N9 viruses did not replicate in the lungs of 4-week-old chickens, indicating that systemic infection would not occur in the infected chickens. In general, H7N9 was more attenuated than H7N2 in 4-week-old chickens.

### 3.4. Efficient Seroconversion of Chickens by Direct-Contact Transmission of H7 Viruses 

**Figure 4:**
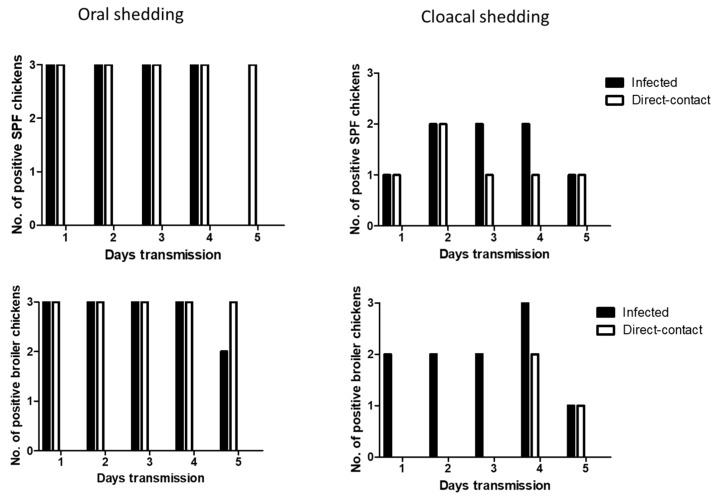
Transmission of H7N2 in SPF and broiler chickens. Three infected birds with each virus (10^7^ pfu/mL) and three contact exposure birds (naïve birds) were placed in an isolator in one day apart. From following day, swab samples were collected daily up to 5 days for virus shedding.

We determined the transmissibility of H7N2 and H7N9 by direct-contact in SPF and broiler chickens. At 1 dpi, oral shedding of H7N2 was detected in all the infected SPF and broiler chickens. After 1 dpe, all the contact exposed SPF and broiler chickens showed oral shedding of H7N2. Viral shedding remained in chickens until 5 dpe (Figure 4). On day 4 post-exposure, infected broiler chickens showed all positive in cloacal shedding. In the contact exposed chickens, more cloacal shedding of H7N2 was detected in SPF chickens than broiler chickens. 

**Figure 5:**
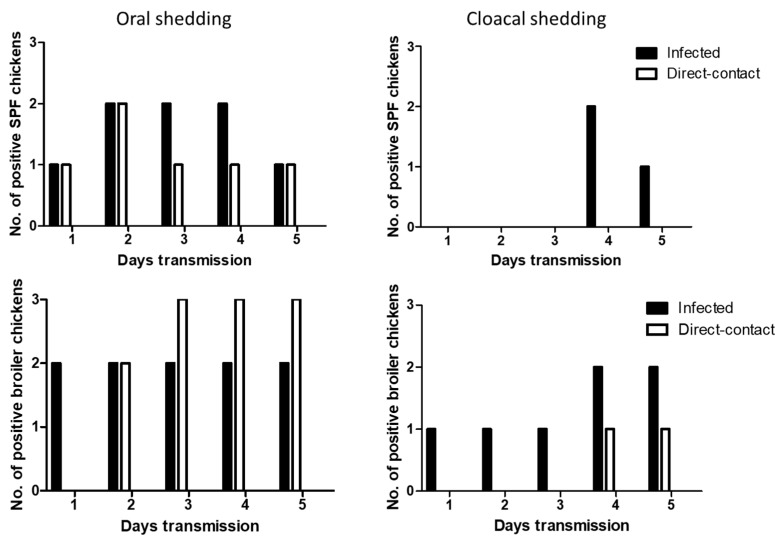
Transmission of H7N9 in SPF and broiler chickens. Three infected birds with each virus (10^7^ pfu/mL) and three contact exposure birds (naïve birds) were placed in an isolator in one day apart. From following day, swab samples were collected daily up to 5 days for virus shedding.

Oral shedding of H7N9 was partially detected in both chickens with maximum positive numbers of two chickens (Figure 5). However, oral shedding of H7N9 was detected in all the direct exposed broiler chickens at 3 dpe, and their shedding remained until 5 dpe. Cloacal shedding of H7N9 was limited in infected SPF and broiler chickens. In direct exposed groups, cloacal shedding was all negative in SPF chickens, and only one broiler chicken showed positive. 

**Figure 6:**
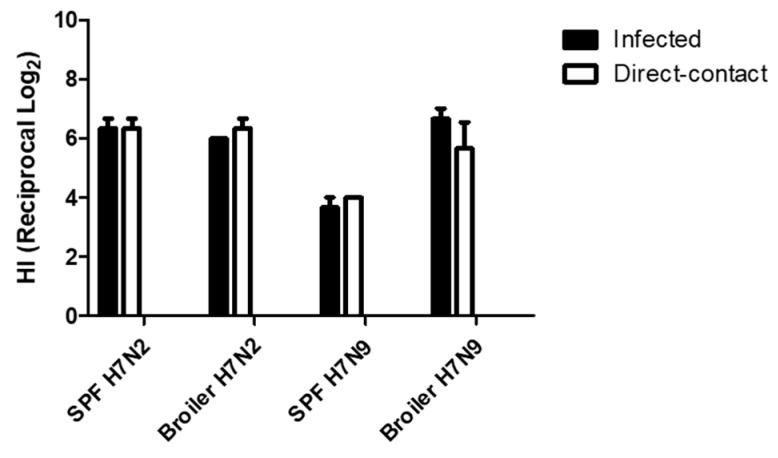
Seroconversion of SPF and broiler chickens. Serum samples were collected from Infected and contact exposure birds at 14 days post-exposure. Seroconversion was determined by HI assay.

All chickens were monitored for exhibition of clinical signs for 14 dpe. They were healthy except for one broiler chicken infected with H7N2. Mortality of the chicken was found at 10 dpi, although we did not find specific lesions associated with avian influenza infection by necropsy. At 14 dpe, serum samples were collected from all the remaining chickens to determine seroconversion. All chickens were seropositive (Figure 6). In H7N2 infected groups, we did not find significant difference in HI tiers (>2^6^) between infected and direct-contact exposed chickens and between SPF and broiler chickens. In contrast, groups of chickens with H7N9 showed different pattern in seroconversion. HI titers of SPF chickens were significantly lower than those of broiler chickens. Interestingly, we found comparable levels of HI titers of broiler chickens infected with H7N9 to those of SPF and broiler chickens infected with H7N2. This suggests that H7N9 might replicate more efficiently in broiler chickens than in SPF chickens. 

## 4. Discussion

LPAI is an economically important disease and remains a continuous threat to commercial poultry operations [17]. H5 and H7 LPAI viruses have been associated with sporadic influenza outbreaks in commercial poultry in North America [18]. LPAI viruses usually cause only a mild or no clinical disease, but may induce enhanced clinical signs in poultry in the field resulting from bacterial co-infections and/or presence of adverse environmental conditions [19]. For example, the outbreaks of H7N9 in turkey breeder flocks resulted in respiratory disease, decreased egg production with limited mortality [17,20]. In chickens, H7N2 infection has caused rapid spread and multi-causal respiratory disease [20]. In contrast, most LPAI infection did not cause detectable clinical signs in SPF chickens. Therefore, we evaluated replication and transmissibility of H7 LPAI viruses in SPF and broiler chickens. We also found that H7N9 replicated more efficiently in broiler chickens than in SPF chickens. 

Our study suggests that H7 LPAI viruses are avirulent in domestic poultry. We further found attenuation of H7N8 and H7N9 compared to H7N2. Multiple outbreaks of LPAI H7N2 occurred in chickens and turkeys between 1996 and 2004 in the Eastern U.S. [21]. However, there is no direct indication that circulation of H7N2 in the fields has resulted in emergence of HPAI virus. Since H7N8 and H7N9 viruses were the precursors of HPAI viruses, the genomic sequence analysis showed 99% of amino acid sequence identity between HPAI and LPAI viruses [12,13]. Their major difference is the HA cleavage site sequence. In the HA of H7N8, the cleavage site sequences are PENPKTRGLF (LPAI) and PENPKKRKTRGLF (HPAI). Similarly, H7N9 HPAI has the insertion sequence at the HA cleavage site (PENPKTDRKSRHRRIRGLF; underline). The change in the cleavage site sequence of HA protein affects pathogenesis of the viruses in poultry [22,23]. In fact, we found 100% mortality of broiler chickens and turkeys after challenge with HPAI H7N8 (unpublished data). This great difference in mortality and morbidity between HPAI and LPAI H7N8 recapitulates an important role of cleavage site sequence of HA protein in viral pathogenesis despite a polygenic trait of AIV virulence for poultry. In addition, H7N8 and H7N9 LPAI viruses were introduced into commercial poultry by spillover from the infected waterfowl [12,13]. It is possible that they may not have been adapted to gallinaceous species. Furthermore, our study showed host restriction of a turkey isolate of H7N8 based on its shedding and induction of seroconversion in turkeys compared to chickens. 

Direct contact is one of the important ways for bird-to-bird transmission of LPAI viruses [19]. In this study, H7N2 was able to transmit efficiently from infected to naïve birds by direct contact on days one post-exposure. Further, oral shedding of H7N2 was persistent in recipient birds. Similarly, we found persistent shedding of H7N9 in recipient broiler chickens up to 5 dpe. Our experimental study, conducted in an isolator setting, cannot completely simulate field conditions (i.e., mass production, stress, and immunosuppression). However, continuous shedding of H7N2 and H7N9 without causing any clinical signs in infected poultry could result in undetected spread of the virus in the field. In addition, circulation of LPAI viruses can lead to seroconversion of broiler chickens (Figure 6). LPAI-induced antibodies might adversely affect protective efficacy of vaccines and controlling the disease in commercial poultry. Inactivated, oil adjuvanted, whole virus vaccines are commonly available vaccines for AIV in countries with endemic AI [24]. Vaccination with inactivated vaccines can be effective generally when the birds are immunologically mature (>3-week-old ages) [25,26]. Our study also indicated that early vaccination (i.e., 1-day-old poults) would be desirable for preventing spread of H7 LPAI viruses based on their efficient and rapid transmission by oral route in 2–4-week-old chickens. The recent outbreaks of H7N8 and H7N9 HPAI viruses have already shown the importance of monitoring circulation of LPAI viruses in the field. Importantly, detection of H5 and H7 LPAI viruses in poultry is notifiable to OIE [19]. Efficient control of LPAI infection will be an important prevention strategy to prevent potential emergence and outbreaks of HPAI viruses in commercial poultry.
